# Supplementary material for: Investigating social development inequality among steel industry workers in Pakistan: A contribution to social development policies
Source: PLoS One. 2021 Jun 10;16(6):e0253082. doi: 10.1371/journal.pone.0253082 (PMC8192002; doi:10.1371/journal.pone.0253082)
Supplement: S1 File — (DOCX) [file pone.0253082.s001.docx]

**Socio-Economic Conditions of Steel Industry workers**

**Title of Industry:**

**Address/Location:**

**Industrial Estate (zone):**

**Type of Industry: a. SME b. Large**

**Function: a. Processing b. Assembling c. Manufacturing**

**No. of Workers: a. <50 b. >50**

**Social Conditions**

1.Gender

1.Male 2.Female 3.Transgender

2. Marital status

a.Married b.Unmarried c.Separated

3. Age Group

a.18-20 b.21-30 c.31-40 d.41-50 e.>50

4. Educational Qualification

a.Illiterate b. Primary c. Middle d. Matric e. Intermediate or above

5. Skill Level

a. unskilled b. semi-skilled c. skilled

6. How many family members you have?

a.1 b.2 c.3 d.4 e.5 f.>5

7. How many dependent members in your household

a.1 b.2 c.3 d.4 e.5 f.>5

8. Job Status

1 daily wages 2. Short term contract (<1year) 3. Medium term contract (1-5years 4. Contract for more than 5 years 5. Permanent (continues till the age of retirement)

9. Job Experience

a.<2years b.2-3years c.3-5years d.5-10years e.>10years

10. Working hours of job

1. <5 hours 2. 5-8 Hours 3. 8-9 Hours 4. 9-10 Hours 5. 10-12 Hours

6. >12Hours

11. Overtime work hours allowed/availed

a.<1 Hour b.1-2 Hours c.2-3Hours d.3-4 Hours e.>4Hours

12. Casual Leave Allowed Annually

a. No leave b. <5days c.5-10days d.10-15days e.>15days

13. Health insurance/ Social security

a. Provided b. Not Provided

14. Accommodation on Job (persons share a room/quarter)

a. Not provided b. 1-3 c. 3-5 d. 5-7 e. >7

15. Distance to job place

a. <1km b. 1-3km c.3-5km d.5-10km e.>10km

16. Transportation facility provided by the Factory

a. Not at all b. one way c. both way d. Conveyance Allowance

17. Commuting to and from Job place

a. Daily b. Weekly c. Fortnightly d. Monthly e. Not specific

18. Safety Measures (Fire Alarms, Emergency Exists, Safety Gears etc)

a. Not Provided b. Unsatisfactory c. Satisfactory

19. First aid treatment at Factory

a. Not Provided b. Unsatisfactory c. Satisfactory

20. Existence of labor union

a. Not allowed b. In-efficient c. Efficient

21. Implication of Labor laws

a. Not followed b. To some extent c. Satisfactory

22. Job security (Hiring and Firing as per labour laws)

a. Not followed b. To some extent c. Satisfactory

23. Working Environment

a. Pathetic b. Very bad c. Bad d. Satisfactory e. Very Good

**Economic Conditions:**

24. Periodical Salary Structure

a. per hour b. per production unit c. Daily d. Weekly e. Monthly

25. Basic salary Structure if you receive monthly salary

a. not declared b. as per cadre

26. If yes, then how much (pkr/month)

a. <1000 b. 1001-5000 c. 5001-10000 d. 10001-20000 e. >20000

27. Total Salary Structure (pkr)

a. <10k b. 10-12k c. 12-15k d. 15-20k e. >20k

28. Mode of salary

a. Cash b. Cheque c. online transfer d. Others

29. Major Expenditure

a. House rent b. Food c. Utilities d. Health e. Education f. others

30. Saving

a. <1000 b. 1000-2000 c. 2000-3000 d. 3000-5000 e. >5000

31. Bonus/ Incentives

a. Not at all b. on Target c. Bi-annually d. annually e. occasionally/festivals

32. Medical Treatment/ Allowance System

a. Not at all b. monthly c. annually d. as per treatment e. other

33. Increments

a. Not at all b. performance based c. annually d. Others
