# Supplementary material for: Investigating social development inequality among steel industry workers in Pakistan: A contribution to social development policies
Source: PLoS One. 2021 Jun 10;16(6):e0253082. doi: 10.1371/journal.pone.0253082 (PMC8192002; doi:10.1371/journal.pone.0253082)
Supplement: Attachment — Submitted filename: Review Report_PONE-D-21-05693.docx [file pone.0253082.s002.docx]

**Title:** Investigating social development inequality among steel industry workers in Pakistan:  A contribution to social development policies

#### Manuscript ID: PONE-D-21-05693

#### Referee Recommendation: MINOR REVISIONS

I appreciate the author(s) for studying a sensitive issue of social inequality among workers of steel industry in Pakistan and policy formulations for betterment of targeted population. However, I have noted some issues which may render further quality to the paper, as following:

**Title:** The title is ok.

**Abstract:** The abstract has been nicely composed. However, there must be few lines on data collection tool and exact studied population i.e., name of development zone.

**Introduction:** This section has been nicely drafted. However, I would like to recommend the author(s) to update this section via citing recent literature along with consistent formatting for citing these studies. Secondly, the section can be further improved grammatically via good academic writing.

**Study objectives and significance:** This section is nicely written, just need to follow the practice of good academic writing through rephrasing few sentences of this section.

**Material and Methods:** The research design, data collection technique and sampling method are appropriate, and the section has been nicely elaborated. There is a just need to check for formatting and grammatical mistakes.

**Empirical analysis:** The author has nicely explained the results along with appropriate model selection. I am just wondering how did the author check the independence of four categories of dependent variable!!

**Conclusions and recommendations:** This section has been nicely drafted.
