# Supplementary material for: Investigating social development inequality among steel industry workers in Pakistan: A contribution to social development policies
Source: PLoS One. 2021 Jun 10;16(6):e0253082. doi: 10.1371/journal.pone.0253082 (PMC8192002; doi:10.1371/journal.pone.0253082)
Supplement: Attachment — Submitted filename: Response to Reviewers.docx [file pone.0253082.s003.docx]

**Rebuttal letter for PLOS One**

**Manuscript ID: PONE-D-21-05693**

**Title: Investigating social development inequality among steel industry workers in Pakistan: A contribution to social development policies**

**Journal Requirements:**

| **No.** | **Comments** | **Response** |
| --- | --- | --- |
| 1 | Please ensure that your manuscript meets PLOS ONE's style requirements | We ensure that our manuscript meets PLOS ONE's style requirements |
| 2 | Please include additional information regarding the survey or questionnaire used in the study and ensure that you have provided sufficient details that others could replicate the analyses. For instance, if you developed a questionnaire as part of this study and it is not under a copyright more restrictive than CC-BY, please include a copy, in both the original language and English, as Supporting Information. | A copy of Questionnaire form is attached |
| 3 | Please amend your current ethics statement to include the full name of the ethics committee/institutional review board(s) that approved your specific study | Ethics statement is revised in the Methods section of the manuscript with full name of ethics committee/institutional review board(s) that approved our study |
| 4 | Please provide an amended Funding Statement regarding the Role of Funders in your study | An amended Funding Statement is provided in the revised cover letter |
| 5 | Please provide an updated Competing Interests Statement | An updated Competing Interests Statement is provided in the revised cover letter |
| 6 | We note you have included a table to which you do not refer in the text of your manuscript. Please ensure that you refer to Table 3 in your text; if accepted, production will need this reference to link the reader to the Table. | Table 3 incorporated |

**Review Comments to the Author**

**Reviewer 1**

**Referee Recommendation: MINOR REVISIONS**

| **No.** | **Comments** | **Response** |
| --- | --- | --- |
|  | Title: The title is ok. | Thanks, we acknowledge and appreciate your comment |
| 1 | Abstract: The abstract has been nicely composed. However, there must be few lines on data collection tool and exact studied population i.e., name of development zone. | Incorporated as per suggestion |
| 2 | Introduction: This section has been nicely drafted. However, I would like to recommend the author(s) to update this section via citing recent literature along with consistent formatting for citing these studies. Secondly, the section can be further improved grammatically via good academic writing. | Incorporated as per suggestion |
| 3 | Study objectives and significance: This section is nicely written, just need to follow the practice of good academic writing through rephrasing few sentences of this section. | Sentences have been rephrased to shape the document more professional and academic |
| 4 | Material and Methods: The research design, data collection technique and sampling method are appropriate, and the section has been nicely elaborated. There is a just need to check for formatting and grammatical mistakes. | Formatting and grammatical mistakes are checked once again and possible correction have been made |
| 5 | Empirical analysis: The author has nicely explained the results along with appropriate model selection. I am just wondering how the author checked the independence of four categories of dependent variable!! | This study used quintiles of socio-economic index, which is the proxy of household well-being and incomes, such as household assets, material resources and housing characteristics. This statistical approach is part of the logistic regression family and is a simple approach for generalizing binary variables. These quintiles categories are independence of observations. Methodological section rephrased according to the comment. |
| 6 | Conclusions and recommendations: This section has been nicely drafted. | Thanks, we acknowledge and appreciate your comment |

**Reviewer #2:**

**Referee Recommendation: MINOR REVISIONS**

| **No.** | **Comments** | **Response** |
| --- | --- | --- |
| 1 | Make the abstract more concrete and informative about key findings. | Incorporated as per suggestion |
| 2 | Introduction section needs substantial revisions to present the research problem, research gap and the contribution of this study. The author(s) should carry out a thorough literature survey of papers published in a range of top journals in the last three/four years relating to the topic addressed in your manuscript. | Incorporated as per suggestion |
| 3 | Methodology Section talks about the methodology and attributes of the data collection and sample size. The important component, which is neglected here, is the justification of the chosen variables. | Incorporated under heading of “description of explanatory variables”.  The selection of explanatory variables based on the Sustainable Livelihood Framework (SLF). These explanatory variables reflect the worker’s endowments of the different forms of material and social well-being variables such as age, education, job status, skill level, job experience, dependency ratio, health insurance and transport facility etc |
| 4 | The authors used four categories of socioeconomic index, which they claim, are independent to each other. Please justify how are they independent? | This study used quintiles of socio-economic index, which is the proxy of household well-being and incomes, such as household assets, material resources and housing characteristics. This statistical approach is part of the logistic regression family and is a simple approach for generalizing binary variables. These quintiles categories are independence of observations. Methodological section rephrased according to the comment. |
| 5 | Their definitions of four probabilities are also not consistent with each other. | Incorporated as per suggestion |
| 6 | The authors need to cite references in their Empirical Methodology. | Incorporated as per suggestion |
| 7 | The authors present their socioeconomic score against two types of industrial units i.e. SME and large in Figure 1 but do not provide their classification criteria. | Classification criteria of industry has addressed now in table under heading “description of explanatory variables” |
| 8 | The authors need to cite sources of figures and tables. | Incorporated as per suggestion |
| 9 | The authors need to provide proper socio-economic reasoning of their empirical results. | Understand the value of advantages and disadvantages across different groups. These kinds of decomposition analysis help formulate equitable policies. Social development is not simply a matter of physical resources. It is the reality of life that deprived person cannot maintain a proper standard of living. On the other hand, employment or income is not necessarily to end social exclusion between low and high-wage workers. Social exclusion exists due to market and infrastructure failure in industry (Saith, 2001) (Hameed & Qasir, 2019). As a result of these inequities, this study used the socio-economic reasoning of their empirical findings. |
| 10 | The conclusions of this study are very general in nature. If so then such conclusion will not make any sense. Make the section more concrete with viable suggestions for policy. | Conclusion findings have been rewritten as per suggestion. |
